# A Diboronic Acid-Based Fluorescent Sensor Array for Rapid Identification of Lonicerae Japonicae Flos and Lonicerae Flos

**DOI:** 10.3390/molecules29184374

**Published:** 2024-09-14

**Authors:** Ying Bian, Chenqing Xiang, Yi Xu, Rongping Zhu, Shuanglin Qin, Zhijun Zhang

**Affiliations:** 1 School of Pharmacy, Hubei University of Science and Technology, Xianning 437100, China; by@hbust.edu.cn (Y.B.); 15195910708@163.com (C.X.); 2 Xianning Public Inspection and Testing Center, Xianning 437100, China; xuyi15374583664@sina.com (Y.X.); zmmly2012@126.com (R.Z.); 3 Research Center for Precision Medication of Chinese Medicine, FuRong Laboratory, Hunan University of Chinese Medicine, Changsha 410000, China

**Keywords:** fluorescent sensor array, machine learning, Lonicerae japonicae flos, Lonicerae flos, rapid identification

## Abstract

Lonicerae japonicae flos (LJF) and Lonicerae flos (LF) are traditional Chinese herbs that are commonly used and widely known for their medicinal properties and edibility. Although they may have a similar appearance and vary slightly in chemical composition, their effectiveness as medicine and their use in clinical settings vary significantly, making them unsuitable for substitution. In this study, a novel 2 × 3 six-channel fluorescent sensor array is proposed that uses machine learning algorithms in combination with the indicator displacement assay (IDA) method to quickly identify LJF and LF. This array comprises two coumarin-based fluorescent indicators (ES and MS) and three diboronic acid-substituted 4,4′-bipyridinium cation quenchers (Q1–Q3), forming six dynamic complexes (C1–C6). When these complexes react with the ortho-dihydroxy groups of phenolic acid compounds in LJF and LF, they release different fluorescent indicators, which in turn causes distinct fluorescence recovery. By optimizing eight machine learning algorithms, the model achieved 100% and 98.21% accuracy rates in the testing set and the cross-validation predictions, respectively, in distinguishing between LJF and LF using Linear Discriminant Analysis (LDA). The integration of machine learning with this fluorescent sensor array shows great potential in analyzing and detecting foods and pharmaceuticals that contain polyphenols.

## 1. Introduction

Lonicerae japonicae flos (LJF) and Lonicerae flos (LF) have been used as traditional Chinese medicine for their heat-clearing and detoxifying effects for centuries in China, serving as both dietary and medicinal substances [1,2]. They are widely applied in the pharmaceutical, food, and healthcare industries [2]. LJF and LF are closely related species with similar chemical compositions and pharmacological activities. Both LJF and LF consist primarily of organic acids, flavonoids, triterpenoid saponins, iridoid glycosides, and volatile oils in terms of their chemical compositions [3,4]. LJF exhibits several pharmacological activities including anti-inflammatory, antipyretic, antiviral, antibacterial, antioxidant, antitumor, hepatoprotective, and pulmoprotective properties. LF, in contrast, shows antibacterial, anti-inflammatory, antioxidant, hepatoprotective, antitumor, immunomodulatory, and anti-atherosclerotic characteristics [5]. Although LJF and LF share some similarities, they differ significantly in other important features. LJF has a greater concentration of flavonoids, but LF demonstrates clearly higher concentrations of phenolic acids and saponins [3,6]. Notably, the clinical mixing or adulteration of LF with LJF is a major safety concern due to the fact that saponins can cause hemolysis when injected [7]. The term “LF” was used interchangeably with “LJF” until the 2005 edition of the *Chinese Pharmacopoeia* where a clear distinction was made between the two. According to the new edition, LJF refers exclusively to the extract from *Lonicera japonica* Thunb., while LF encompasses the extracts from *L. macranthoides*, *L. hypoglauca*, *L. confusa*, and *L. fulvotomentosa* [8].

In recent years, with the constant development and widespread application of LJF resources and industry, over 500 Chinese patented medications contain LJF, such as Lianhua Qingwen capsules, Jinyinhua dew, and Jinyinhua mixture [9,10]. During the outbreak of SARS in 2003 and the COVID-19 pandemic in 2019, LJF was extensively used as a primary antiviral drug [11]. With the increasing recognition given to and the promotion of traditional Chinese medicine worldwide, there is a growing demand for LJF. Unscrupulous sellers have taken advantage of the close physical resemblance between LJF and LF by fraudulently marketing the cheaper LF as the more expensive LJF [12]. This has resulted in unfair competition and economic losses in the medicinal market. Therefore, it is imperative to develop precise, effective, and accessible techniques for swiftly distinguishing LJF from LF. This is crucial to ensuring the safety of medication, supporting market regulation, and advancing the Chinese medicinal material identification technology.

It has been demonstrated in previous studies that the methods used to identify LJF and LF primarily focus on morphological, microscopic, spectroscopic, chromatographic, and biotechnological techniques. It is difficult to accurately distinguish between LJF and LF through traditional morphological and microscopic identification because of the nuanced variations, which necessitates a high level of skill. Kang et al. [13] conducted a study on the pharmacognostic features of LJF and LF by identifying the original plants and their traits, and analyzing them under microscope. Yan et al. [14] established an approach for infrared spectroscopic tri-step identification where Fourier-transform infrared (FT-IR) spectroscopy and two-dimensional correlation analysis were performed to rapidly identify LJF and LF. By using the PCA-LDA model, He et al. [15] detected LJF and LF and located their geographical origins with a 100% accuracy by integrating chemometrics with excitation–emission matrix fluorescence (EEMF). Wang et al. [16] performed near-infrared hyperspectral imaging (HSI) to create support vector classification (SVC) models based on a linear kernel function. These models achieved an accuracy of 98.46–100% in classifying the species of LJF and LF. Cai et al. [17] performed ultra-fast liquid chromatography–triple quadrupole/linear ion trap mass spectrometry (UFLC-QTRAP-MS/MS) in conjunction with multivariate statistical analysis to obtain comprehensive information for the quality control of LJF and LF. In their study, Wu et al. [18] distinguished between LJF and LF by analyzing volatile organic compounds (VOCs) in the former through multivariate statistics in combination with headspace gas chromatography–ion mobility spectrometry and headspace solid-phase microextraction gas chromatography–mass spectrometry. Liu et al. [12] applied ^1^H-NMR in combination with chemometric pattern recognition to generate characteristic fingerprint spectra, successfully classifying and identifying LJF and LF. The LDA models yielded a predictive accuracy of 95.65% and 98.1% for LJF and LF, respectively. In order to facilitate the quantitative and qualitative examination of LJF samples that had been intentionally mixed, Gao et al. [19] applied DNA metabarcoding technology to create a short mini-barcode primer in the psbA-trnH region. Wang et al. [20] combined loop-mediated isothermal amplification with cationic conjugated polymer to efficiently differentiate between LJF and LF by means of SNP genotyping. However, these methods face various challenges such as the need for expensive instruments, the high complexity in sample preparation, lengthy analysis, and proficiency in operation. As far as we know, there is currently no technology that combines the cross-reactivity principle of fluorescent array sensors with machine learning to differentiate between LJF and LF in a straightforward, quick, efficient, and cost-effective way.

To identify LJF and LF rapidly, a 2 × 3 six-channel sensor array was created using machine learning techniques in conjunction with the indication displacement assay (IDA) technique [21,22,23]. Two coumarin-based fluorescent indicators (ES and MS) and three diboronic acid-substituted 4,4′-bipyridinium cations (Q1−Q3) comprise the array, with six dynamic complexes (C1–C6) formed. In addition, the polyhydroxy phenolic acid components of LJF and LF interact with these complexes to induce different fluorescence responses. Linear Discriminant Analysis (LDA) was conducted to quickly differentiate between LJF and LF after distinct “fingerprint patterns” were developed using machine learning techniques [24,25].

Furthermore, tests were conducted on four main phenolic acids and 16 batches of samples derived from LJF and LF to validate the identification mechanism and sensing capability of the fluorescent array. The results indicated that the sensor array could distinguish between LJF and LF rapidly and accurately by recognizing phenolic acids with ortho-diphenol hydroxyl groups. In order to evaluate the capability of the fluorescent array in distinguishing the complex systems containing phenolic acid components and displaying subtle variations, it was used to differentiate commercially available Jinyinhua mixtures. This resulted in the successful identification of products obtained from three distinct manufacturers with a high level of accuracy. In summary, this method is simple, fast, highly sensitive, and accurate, contributing an efficient solution to distinguishing between LJF and LF.

## 2. Results and Discussion

### 2.1. Principle of Fluorescent Sensor Array Response

**Figure 1:**
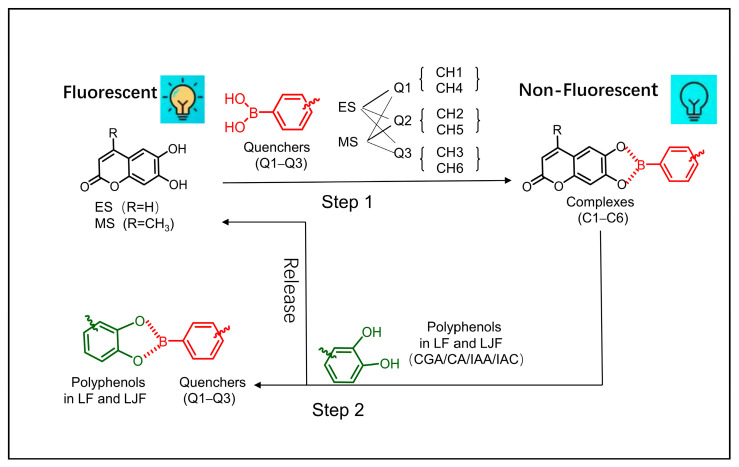
Schematic diagram of response from the fluorescent sensor array.

The chemical composition of traditional Chinese medicinal materials is diverse and complex. The main components of both LJF and LF include phenolic acids, flavonoids, saponins, and sugars [5]. Phenolic acids, such as isochlorogenic acid A (IAA), isochlorogenic acid C (IAC), caffeic acid (CA), and chlorogenic acid (CGA), could be taken as indicators of LJF quality [26]. A 2 × 3 six-channel fluorescent sensor array was built in this study through a combination of spatial distance regulation and the indicator displacement assay (IDA) mechanism [24]. This array comprises two fluorescent indicators (6,7-dihydroxy-4-methylcoumarin, ES, and 6,7-dihydroxycoumarin, MS) and three quenchers (Q1−Q3), forming six dynamic complexes (C1–C6). The formation of these dynamic complexes quenched the fluorescence intensity of the system (Figure 1; Step 1). In LJF and LF, the main phenolic acids possess ortho-dihydroxy structures that can interact with the boric acid structure and selectively bind the quenchers (Q1–Q3) to create stable five-membered pseudocycles [27,28]. This competitive binding releases the fluorescent indicators (ES and MS), leading to varying degrees of fluorescence recovery (Figure 1; Step 2). The ortho-dihydroxy phenolic acids bind selectively with the quenchers, leading to differential fluorescence recovery (Supplementary Figure S4), because of the structural variations in Q1–Q3. The differences in the type and concentration of phenolic acids in LJF and LF lead to differential fluorescence responses when they are introduced to the fluorescent sensor array. This fact enables the rapid identification and differentiation of LJF and LF.

### 2.2. Determination of Concentration of Q1–Q3 Using Fluorescence Quenching Curves

**Figure 2:**
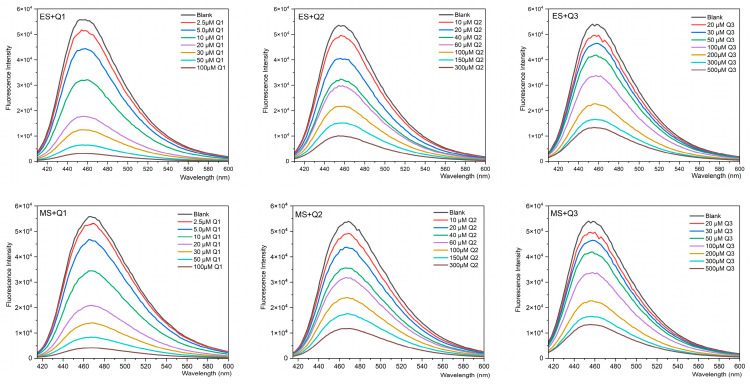
Fluorescence quenching curves of ES (8 μM) and MS (5 μM) titrated with different concentrations of the quenchers (Q1–Q3) in PBS (pH 7.2–7.4).

As shown in Figure 2, the fluorescence intensity of the indicators was retained at 30% when the concentration of Q1–Q3 was 30 μM, 150 μM, and 300 μM, respectively. It was found that these concentrations were optimal for building the sensor array [21].

### 2.3. Quenching Curves for Differentiation of 4 Types of Phenolic Acid Components by Fluorescent Sensors

**Figure 3:**
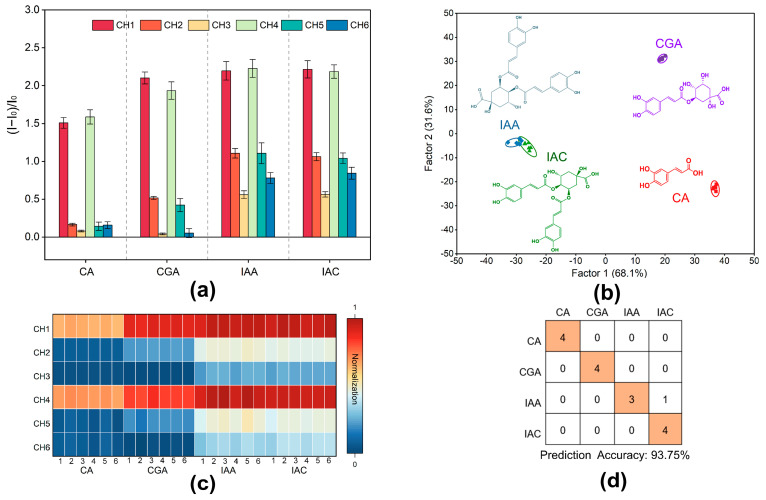
Identification of four phenolic acids by the designed sensor array. (**a**) The response of the 6-channel sensor array to four phenolic acids (CH = channel); the error bar represents the standard deviation of 6 replications. (**b**) LDA classical score map of fluorescence response to the 4 phenolic acids obtained by the sensor array (scores were generated by LDA with 95% confidence). (**c**) Heat map of the fluorescence response of the multichannel array sensor to the four phenolic acids. (**d**) Confusion matrix plots of 16 unknown samples of the four phenolic acids.

A test was conducted on the four main phenolic acids (IAA, IAC, CA, and CGA; 50 μM) found in these plants to better understand the mechanism of fluorescence response in the LJF and LF samples. The fluorescence response to the four phenolic acids varied across the six channels of the array, as shown in Figure 3a. The heat map in Figure 3c shows the different patterns of fluorescence response from the constructed array exposed to the four different components of phenolic acid. Because of the spatial configuration that enables Q1 to bind more effectively to the phenolic acids, which leads to considerable fluorescence recovery and the release of more ES and MS, the most dramatic responses were observed in Channels 1 and 4. In comparison, Q2 and Q3 were not as effective in binding, which leads to the less significant release of ES and MS and poorer fluorescence recovery. IAA and IAC, both of which contain two ortho-diphenol hydroxyl recognition sites, exhibited more significant changes in fluorescence response compared to CGA and CA, which contain only one such recognition site. In contrast to CGA and CA, which have only one ortho-diphenol hydroxyl recognition site, the change in fluorescence response modifications was more pronounced in IAA and IAC because they contain two of these sites each. Since IAA and IAC have identical molecular structures and the same number of recognition sites, there is little variation in their response.

In order to assess the sensor array for its capability of recognition, an LDA training matrix model with six channels, four phenolic acids, and six repetitions was constructed. Different types of phenolic acids were clearly divided into four distinct clusters, as shown in Figure 3b, which reveals that the phenolic acids clustered into one category. In the LDA analysis, the two most important variables, Factor 1 and Factor 2, explained 69.30% and 30.40% of the total variance, respectively, which amounted to 99.70% in total (Figure 3b). Topping the charts with a 91.50% accuracy was the LDA Jackknifed classification matrix (Supplementary Tables S1 and S2). For validation, 16 unknown samples of the four phenolic acids were randomly selected as the samples of the blind test. The accuracy was 93.75%, with 15 samples correctly predicted (Figure 3d; Supplementary Table S3). A phenolic acid molecule with an ortho-diphenol hydroxyl structure was detected by the fluorescent array sensor, according to these findings. These results suggest that the constructed fluorescent array sensor is applicable to the identification of LF and LJF.

### 2.4. Identification of 14 Baches of LJF and LF 

**Figure 4:**
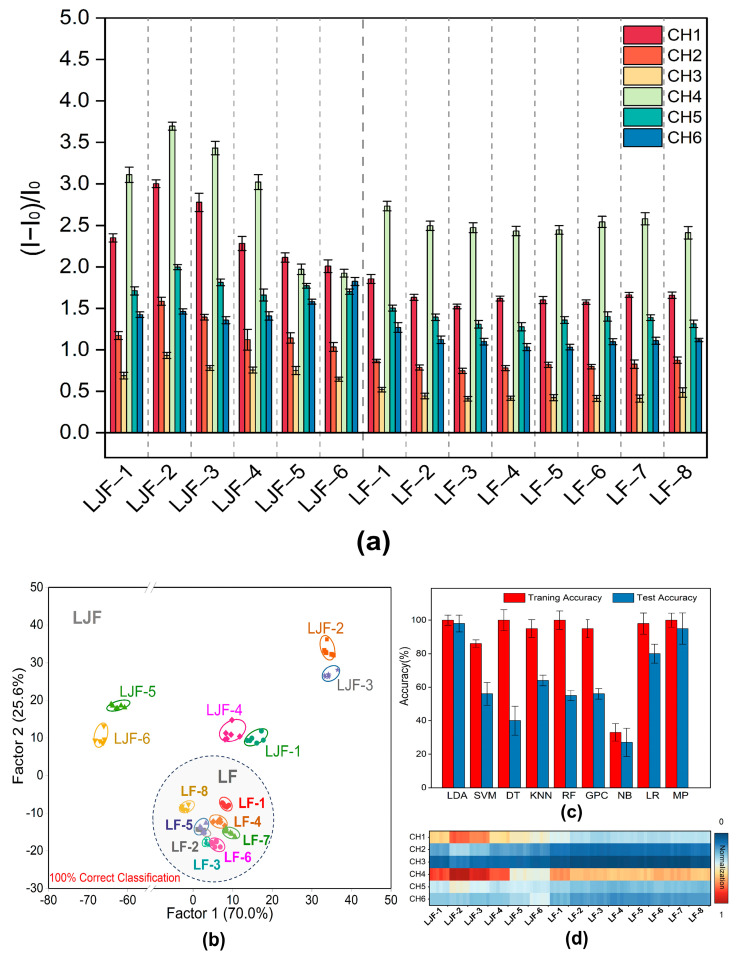
Identification of 14 baches of LJF and LF by the designed sensor array. (**a**) The response of the 6-channel sensor array to 14 baches of LJF and LF (CH = channel). The error bar represents the standard deviation of 6 tests. (**b**) LDA classical score plot of fluorescence response for the 14 samples obtained by the sensor array. (**c**) Heat map of the fluorescence response of the multichannel array sensor to 14 baches of LJF and LF. (**d**) Accuracy of training and test of the data on fluorescence response of the array sensors for the 14 baches of LJF and LF by 9 machine learning algorithms.

In total, 14 batches of LJF and LF samples were purchased from the market and tested randomly to establish how well the sensor array could identify them. Channels 1 and 4 exhibited the most noticeable responses among the six channels (Figure 4a), which is consistent with the patterns of fluorescence response for phenolic acids (Figure 3a). Meanwhile, significant differences were observed across the six channels (Figure 4c).

Figure 4d shows the results of data optimization and application of nine distinct machine learning techniques to improve the accuracy of LJF/LF classification, namely Linear Discriminant Analysis (LDA), Support Vector Machine (SVM), Decision Tree (DT), k-Nearest Neighbors (KNN), Random Forest (RF), Gaussian Process Classifier (GPC), Naive Bayes (NB), Logistic Regression (LR), and Multi-Layer Perceptron (MP). The results indicated that among these algorithms, Linear Discriminant Analysis (LDA) outperformed all the other algorithms in terms of predictive performance, with an accuracy of 100% and 98% reached in the training and prediction phases, respectively. Afterwards, a reliable training model was built through Linear Discriminant Analysis (LDA) of the data. Canonical scores were generated using a training matrix that consisted of six channels, 14 samples, and six repetitions to evaluate the identification performance for the 14 batches of LJF and LF. According to the LDA plot, Factor 2 contributed to 25.6% of the total variance, while Factor 1 accounted for 70.0% of the total variance, which illustrates the best discriminative ability. As shown in Figure 4b, Factors 1 and 2 accounted for 95.6% of the overall variation. Eight batches of LF samples clustered in the lower part of the LDA plot (gray circle in Figure 4b), which clearly distinguishes them from the six batches of LJF samples. It is shown that the Jackknife classification matrix achieved a 100% accuracy of classification (Supplementary Tables S4 and S6). To verify the predictive performance for unknown LJF and LF samples, 55 unknown samples were added to the sensor array. The array achieved a predictive accuracy of 98.21% by correctly predicting all 56 samples (Supplementary Table S5). This proves that the constructed fluorescent sensor array is capable of differentiating between LJF and LF accurately.

### 2.5. Discrimination of Jinyinhua Mixtures from Three Different Manufacturers

**Figure 5:**
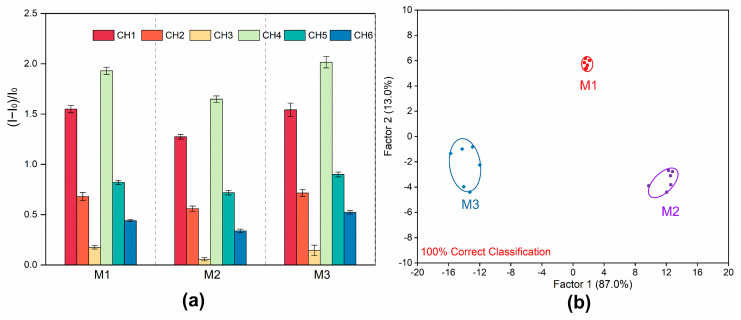
Identification of Jinyinhua mixtures from 3 different manufacturers. (**a**) The response of the 6-channel sensor array to Jinyinhua mixtures from 3 different manufacturers. (CH = channel); the error bar represents the standard deviation of 6 replications. (**b**) LDA classical score map of fluorescence response for the Jinyinhua mixtures from 3 different manufacturers obtained by the sensor array.

It is widely known that Jinyinhua mixtures made by different manufacturers with LJH from different producing areas vary slightly in taste and efficacy. Therefore, the three most popular Jinyinhua mixtures from various manufacturers were selected to test the fluorescent array on its capacity to identify these complex systems rich in phenolic acid components. For these mixtures, a training matrix with six channels, 14 samples, and six repetitions was created. Then, the data were converted into typical scores. The Jinyinhua mixtures from three manufacturers were clearly distinguished in the LDA results (Figure 5a,b), with a 100% accuracy demonstrated for sample differentiation by the Jackknife classification matrix (Supplementary Tables S7 and S9). To verify the predictive performance for unknown Jinyinhua mixtures, 12 unknown samples were added to the sensor array, with a predictive accuracy of 100% achieved by correctly predicting all 12 samples (Supplementary Table S8). To sum up, the phenolic acid compound-rich complex systems can be quickly detected using the fluorescent array proposed in this study. Not only does it reliably distinguish between LJF and LF, but it also performs well in differentiating between the Jinyinhua mixtures produced by different manufacturers.

## 3. Materials and Methods

### 3.1. Instruments

The equipment used in this study included Bruker Avance III 400 MHz Nuclear Magnetic Resonance Spectrometer (Bruker, Ettlingen, Germany), Motic-M200 Digital Interactive Microscope (Motic Industrial Group Co., Ltd., Xiamen, China), Tecan Spark 10M Full Wavelength Multi-function Microplate Reader (Tecan, Uster, Switzerland), FA2004N Analytical Balance (Shanghai Jinghai Instrument Co., Ltd., Shanghai, China), Ultrasonic Cleaner (Hunan Shangxinchuang Instrument Equipment Co., Ltd., Changsha, China), and WHB-96CB-B Microplate (Shanghai Wohong Biotechnology Co., Ltd., Shanghai, China).

### 3.2. Reagents

The reagents used in this study included isochlorogenic acid A (IAA) (National Institutes for Food and Drug Control, Kanagawa, Japan), isochlorogenic acid C (IAC) (National Institutes for Food and Drug Control), caffeic acid (CA) (Shanghai Jizhi Biochemical Technology Co., Ltd., Shanghai, China), chlorogenic acid (CGA) (Shanghai Bide Pharmaceutical Technology Co., Ltd., Shanghai, China),6,7-Dihydroxy-4-methylcoumarin (ES) (Shanghai Yuanye Bio-Technology Co., Ltd., Shanghai, China), 6,7-Dihydroxycoumarin (MS) (Shanghai Macklin Biochemical Co., Ltd., Shanghai, China), 2-Bromomethylphenylboronic acid (Shanghai Haohong Biopharmaceutical Co., Ltd., Shanghai, China), 3-Bromomethylphenylboronic acid (Shanghai Jizhi Biochemical Technology Co., Ltd., Shanghai, China), 4-Bromomethylphenylboronic acid (Shanghai Bide Pharmaceutical Technology Co., Ltd., Shanghai, China), N,N-Dimethylformamide, 99.8% (DMF) (Shanghai Yien Chemical Technology Co., Ltd., Shanghai, China), 4,4′-Bipyridine (Shanghai Aladdin Biochemical Technology Co., Ltd., Shanghai, China), PBS phosphate buffer (dry powder) (Beijing Lanjie Ke Technology Co., Ltd.), Jinyinhua mixture (M1) (Beihai Gofar Chuanshan Biological Co., Ltd., Beihai, China), Jinyinhua mixture (M2) (Guangxi Bangqi Pharmaceutical Group Co., Ltd., Qinzhou, China), and Jinyinhua mixture (M3) (Guangxi Kangsheng Pharmaceutical Co., Ltd., Nanning, China).

### 3.3. Sample Collection and Identification

In total, 14 batches of LJF and LF samples were purchased from the traditional Chinese medicinal markets in Bozhou, Anguo, and other regions. The botanical origins of the 14 batches of medicinal materials were determined according to the morphological identification and microscopic characteristics of corolla surface slices (Table 1). The information about these samples is shown in Table 2. The samples were stored in the Chinese medicinal specimen room affiliated with the School of Pharmacy at Hubei University of Science and Technology.

### 3.4. Sample Preparation

After being ground, the 14 batches of samples were filtered through a 60-mesh sieve, and 0.05 g of the powdered medicinal material was added to 10 mL of ultrapure water. After 5 min of ultrasonic extraction, the mixture was filtered. The resulting filtrate was centrifuged at 8000 r/min for 10 min. Finally, the supernatant was stored at 4 °C for later use.

To prepare the solution of Jinyinhua mixtures as sample, 1 mL of the Jinyinhua mixtures was taken and diluted with PBS to a volume of 25 mL, mixed thoroughly, and stored at 4 °C for later use.

### 3.5. Synthesis of Quenchers (Q1–Q3)

**Figure 6:**
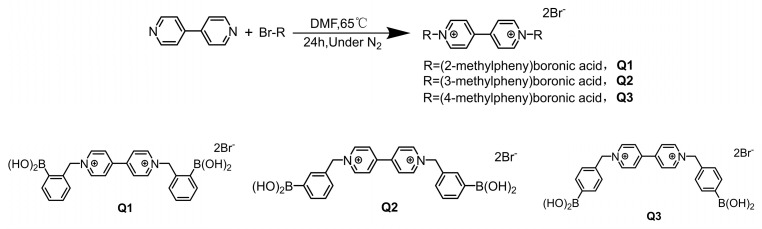
The route of synthesis of Q1–Q3.

A white solid product (326 mg, yield 86.9%) was obtained by mixing 2-(bromomethyl) phenylboronic acid (345 mg, 1.6 mmol) and 4,4′-bipyridine (100 mg, 0.64 mmol) in 3 mL volume of anhydrous N,N-dimethylformamide. The mixture was stirred at 65 °C in the presence of nitrogen for 24 h, cooled to room temperature, and filtered. Then, the precipitate was washed multiple times with acetone. The synthetic was Q1. The structure of the compound was confirmed as ortho-bromomethyl phenylboronic acid-substituted 4,4′-bipyridinium cations [29] by ^1^H-NMR (400 MHz, DMSO-d6) *δ*: 6.16 (4H, s), 7.37 (2H, dd, *J* = 8.0, 2.0 Hz), 7.48 (4H, m), 7.85 (2H, dd, *J* = 8.0, 2.0 Hz), 8.75 (4H, d, *J* = 8.0 Hz), 9.33 (4H, d, *J* = 8.0 Hz). Q2 and Q3 were synthesized in a similar way by replacing 2-(bromomethyl) phenylboronic acid with 3-(bromomethyl) phenylboronic acid and 4-(bromomethyl) phenylboronic acid, respectively. Figure 6 depicts the pathway of synthesis of Q1–Q3.

### 3.6. Optimization of Q1–Q3 Concentration and Construction of Fluorescent Sensor Array

Fluorescence spectra were recorded when the emission wavelength ranged from 415 to 600 nm. The excitation wavelength of ES and MS was set to 356 nm and 368 nm, respectively. To assemble the sensor array, varying amounts of Q1–Q3 were added into either the ES (8 μM) or MS (5 μM) solution. The quencher concentration that reduces the fluorescence of the sensing system to 30% was chosen. To obtain 100 μL of the sensor solution (ES-Q1, ES-Q2, ES-Q3, MS-Q1, MS-Q2, MS-Q3), 50 μL of each fluorescent indicator (ES; MS) was combined with 50 μL of each quencher (Q1–Q3). This facilitated the creation of the fluorescent sensor array.

### 3.7. Determination of Four Phenolic Acid Components

To prepare 50 μM of the solutions, the appropriate amounts of IAA, IAC, CA, and CGA were diluted with PBS buffer. Then, 100 μL of each phenolic acid solution was added with 100 μL of the sensor solution in a 96-well plate, and the intensity of fluorescence was measured using a SPARK microplate reader. The change in fluorescence intensity is expressed as (I − I_0_)/I_0_, where I and I_0_ represent the fluorescence intensity with and without the addition of the four phenolic acids, respectively. All experiments were conducted in 0.01 mM PBS phosphate buffer at a pH value ranging from 7.2 to 7.4.

### 3.8. Determination of 14 Batches of Samples and Jinyinhua Mixtures from 3 Different Manufacturers

A SPARK microplate reader was used to measure the intensity of fluorescence after 100 μL of the samples was thoroughly mixed with 100 μL of the sensor solution in multiple black 96-well plates. The formula used to calculate the change in fluorescence intensity is expressed as (I − I_0_)/I_0_, where I and I_0_ are the fluorescence intensity measured before and after the addition of the samples, respectively.

### 3.9. Data Analysis

All data were processed using Origin 2022, Sysyat 13.0, and Python 3.8. The machine learning models were derived from scikit-learn (https://scikit-learn.org/stable/index.html, accessed on 26 June 2024). The optimization of algorithms and processing of data were carried out through nine machine learning algorithms, which were Linear Discriminant Analysis (LDA), Support Vector Machine (SVM), Decision Tree (DT), k-Nearest Neighbors (KNN), Random Forest (RF), Gaussian Process Classifier (GPC), Naive Bayes (NB), Logistic Regression (LR), and Multi-Layer Perceptron (MP).

## 4. Conclusions

This study used two coumarins with ortho-dihydroxy structure, namely 6,7-dihydroxy-4-methylcoumarin (ES) and 6,7-dihydroxycoumarin, (MS), as fluorescent indicators by employing the indicator displacement assay (IDA) methodology. Three diboronic acid-substituted 4,4′-bipyridinium cations (Q1–Q3) were synthesized as fluorescence quenchers through the reaction of bromomethylphenylboronic acids at the ortho, meta, and para positions with 4,4′-bipyridine. In order to rapidly identify LJF and LF, a 2 × 3 array of six-channel fluorescence sensors was built. This simple, rapid, and efficient method was used to successfully distinguish 14 batches of LJF and LF samples with a 100% accuracy of classification and a 98.21% accuracy of prediction.

The response mechanism of the fluorescent sensor array for differentiating LJF and LF was inferred through the detection of four types of phenolic acid components. The molecules in LJF and LF have different ortho-dihydroxy structures, which explains why the responses are different. Although the approach achieved an accuracy of only 91.50% when used to differentiate between the four phenolic acid types, it was 100% accurate when used to differentiate between LJF and LF. The potential reasons for the improved accuracy of detection include the presence of additional chemicals with ortho-dihydroxy structure that react with the fluorescent sensor array, leading to stronger fluorescence signals. This proves that the fluorescent sensor array is useful for analyzing intricate systems.

When it comes to the detection of complicated multi-component combinations, fluorescent sensor arrays have distinct advantages [30,31,32]. Their use differs from the conventional “lock-and-key” method as it compares or tracks the variations in the type and content of various components across different samples [33]. Traditional Chinese medicine is known for its diverse and complex components. The design of corresponding fluorescent sensor arrays has potential in distinguishing the near-source varieties of traditional Chinese medicine and authenticating medicinal materials [34,35]. This study also demonstrates that fluorescent sensor array technology can effectively capture the slight differences in chemical components between LJF and LF and visualize these differences through fluorescence signals. This molecule-based identification technique provides new approaches to the study, detection, and quality control of polyphenol-rich foods and natural medicinal products.

## Figures and Tables

**Table 1:** The macroscopic and microscopic characteristics of Lonicerae Japonicae Flos and Lonicerae Flos samples.

Characteristic	LJF	LF		
	*L. japonica*	*L. macranthoides*	*L. hypoglauca*	*L. confusa*
form	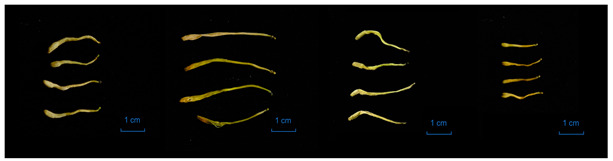			
macroscopic characteristics	yellowish-white or greenish-white, densely covered with greyish-white hairs	greenish-brown or yellowish-white, nearly glabrous	yellowish-white or yellowish-brown, sparse hairs	yellowish-brown, bud slight, densely covered with white hairs
surface view of corolla	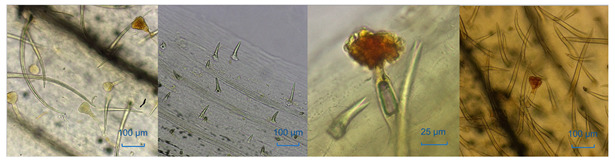			
microscopic characteristics	more spreading non-glandular hairs and glandular hairs	non-glandular hairs a little shorter, sparse glandular hairs	sparse non-glandular hairs, head of glandular hair surrounded with scutellate	more non-glandular hairs, the head of glandular hair sunken in the middle of the top

**Table 2:** Detailed information on the samples of Lonicerae Japonicae Flos and Lonicerae Flos.

Sample No.	Producing Area	Name	Species
LJF-1	Fengqiu, Henan	Lonicerae Japonicae Flos	*L. japonica*
LJF-2	Fengqiu, Henan	Lonicerae Japonicae Flos	*L. japonica*
LJF-3	Pingyi, Shandong	Lonicerae Japonicae Flos	*L. japonica*
LJF-4	Linyi, Shandong	Lonicerae Japonicae Flos	*L. japonica*
LJF-5	Julu, Hebei	Lonicerae Japonicae Flos	*L. japonica*
LJF-6	Tangshan, Hebei	Lonicerae Japonicae Flos	*L. japonica*
LF-1	Anlong, Guizhou	Lonicerae Flos	*L. confusa*
LF-2	Bozhou, Anhui	Lonicerae Flos	*L. macranthoides*
LF-3	Shaoyang, Hunan	Lonicerae Flos	*L. macranthoides*
LF-4	ziyuan, Guangxi	Lonicerae Flos	*L. macranthoides*
LF-5	Bozhou, Anhui	Lonicerae Flos	*L. hypoglauca*
LF-6	Ziyuan, Guanxi	Lonicerae Flos	*L. macranthoides*
LF-7	Xinyang, Henan	Lonicerae Flos	*L. macranthoides*
LF-8	Julu, Hebei	Lonicerae Flos	*L. hypoglauca*

## Data Availability

The original contributions presented in the study are included in the article/Supplementary Material, further inquiries can be directed to the corresponding authors.

## Supplementary Materials

The following supporting information can be downloaded at https://www.mdpi.com/article/10.3390/molecules29184374/s1, Figures S1–S3: the ^1^H NMR spectrum of compound **Q1**–**Q3**; Figure S4, Tables S1–S9: statistical analysis.

## References

[1] Li Y., Li W., Fu C., Song Y., Fu Q. (2020). *Lonicerae japonicae* Flos and Lonicerae Flos: A Systematic Review of Ethnopharmacology, Phytochemistry and Pharmacology. Phytochem. Rev..

[2] Zheng S., Liu S., Hou A., Wang S., Na Y., Hu J., Jiang H., Yang L. (2022). Systematic Review of *Lonicerae japonicae* Flos: A Significant Food and Traditional Chinese Medicine. Front. Pharmacol..

[3] Wang L., Jiang Q., Hu J., Zhang Y., Li J. (2016). Research Progress on Chemical Constituents of *Lonicerae japonicae* Flos. Biomed Res. Int..

[4] Yang Q.-R., Zhao Y.-Y., Hao J.-B., Li W.-D. (2016). Research Progress on Chemical Constituents and Their Differences between *Lonicerae japonicae* Flos and Lonicerae Flos. Chin. Mater. Medica.

[5] Li W., Zhang L., He P., Li H., Pan X., Zhang W., Xiao M., He F. (2024). Traditional Uses, Botany, Phytochemistry, and Pharmacology of *Lonicerae japonicae* Flos and Lonicerae Flos: A Systematic Comparative Review. J. Ethnopharmacol..

[6] Cai Z., Wang C., Chen C., Zou L., Chai C., Chen J., Tan M., Liu X. (2021). Quality Evaluation of *Lonicerae japonicae* Flos and Lonicerae Flos Based on Simultaneous Determination of Multiple Bioactive Constituents Combined with Multivariate Statistical Analysis. Phytochem. Anal..

[7] Gao Y., Hou R., Han Y., Fei Q., Cai R., Qi Y. (2018). Shuang-Huang-Lian injection induces an immediate hypersensitivityreaction via C5a but not IgE. Sci. Rep..

[8] Chinese Pharmacopoeia Commission (2020). Pharmacopoeia of the People’s Republic of China.

[9] Miao H., Zhang Y., Huang Z., Lu B., Ji L. (2019). *Lonicera japonica* Attenuates Carbon Tetrachloride-Induced Liver Fibrosis in Mice: Molecular Mechanisms of Action. Am. J. Chin. Med..

[10] Shang X., Pan H., Li M., Miao X., Ding H. (2011). *Lonicera japonica* Thunb.: Ethnopharmacology, phytochemistry and pharmacology of an important traditional Chinese medicine. J. Ethnopharmacol..

[11] Liang Y.C., Long W.W., Qing Z.J., Rui B.Q., Yuan L.J., Ikhlas K., Rudolf B., An G.D. (2022). Traditional Chinese Medicines against COVID19: A Global Overview. World Chin. Med..

[12] Liu K., Jin Y., Gu L., Li M., Wang P., Yin G., Wang S., Wang T., Wang L., Wang B. (2023). Classification and Authentication of *Lonicerae japonicae* Flos and Lonicerae Flos by Using 1H-NMR Spectroscopy and Chemical Pattern Recognition Analysis. Molecules.

[13] Kang S., Zhang J., Wang Y.D., Han H.Q., Liang S., Lu J., Ma S.C. (2014). Pharmacognostic authentication study for *Lonicerae japonicae* Flos and Lonicerae Flos. Chin. J. Pharm. Anal..

[14] Yan R., Chen J., Sun S., Guo B. (2016). Rapid Identification of *Lonicerae japonicae* Flos and Lonicerae Flos by Fourier Transform Infrared (FT-IR) Spectroscopy and Two-Dimensional Correlation Analysis. J. Mol. Struct..

[15] He S., Long W., Hai C., Chen H., Tang C., Rong X., Yang J., Fu H. (2024). Rapid identification of traditional Chinese medicines (Lonicerae japonicae flos and Lonicerae flos) and their origins using excitation-emission matrix fluorescence spectroscopy coupled with chemometrics. Spectrochim. Acta. A Mol. Biomol. Spectrosc..

[16] Wang J., Cai Z., Jin C., Peng D., Zhai Y., Qi H., Bai R., Guo X., Yang J., Zhang C. (2024). Species Classification and Origin Identification of *Lonicerae japonicae* Flos and Lonicerae Flos Using Hyperspectral Imaging with Support Vector Machine. J. Food Compos. Anal..

[17] Cai Z., Wang C., Zou L., Liu X., Chen J., Tan M., Mei Y., Wei L. (2019). Comparison of Multiple Bioactive Constituents in the Flower and the Caulis of *Lonicera japonica* Based on UFLC-QTRAP-MS/MS Combined with Multivariate Statistical Analysis. Molecules.

[18] Wu T., Yin J., Wu X., Li W., Bie S., Zhao J., Song X., Yu H., Li Z. (2024). Discrimination and Characterization of Volatile Organic Compounds in *Lonicerae japonicae* Flos and Lonicerae Flos Using Multivariate Statistics Combined with Headspace Gas Chromatography–Ion Mobility Spectrometry and Headspace Solid-phase Microextraction Gas Chromatography–Mass Spectrometry Techniques. Rapid Commun. Mass Spectrom..

[19] Gao Y., Zhang X., Wang W., Xing Z., Xu L., Tian X. (2023). Qualitative Identification of *Lonicerae japonicae* Flos in Traditional Chinese Medicine Using Metabarcoding Combined with Specific Mini-Barcodes. Mol. Biol. Rep..

[20] Wang Z., Jiang C., Jin Y., Yang J., Zhao Y., Huang L., Yuan Y. (2023). Cationic Conjugated Polymer Fluorescence Resonance Energy Transfer for DNA Methylation Assessment to Discriminate the Geographical Origins of *Lonicerae japonicae* Flos. J. Agric. Food Chem..

[21] Ni W., Yu Y., Gao X., Han Y., Zhang W., Zhang Z., Xiao W., Hu Q., Zhang Y., Huang H. (2024). Multilocus Distance-Regulated Sensor Array for Recognition of Polyphenols via Machine Learning and Indicator Displacement Assay. Anal. Chem..

[22] Xu L., Wang H., Xiao W., Zhang W., Stewart C., Huang H., Li F., Han J. (2023). PAMAM Dendrimer-Based Tongue Rapidly Identifies Multiple Antibiotics. Sens. Actuators B Chem..

[23] Tropp J., Ihde M.H., Williams A.K., White N.J., Eedugurala N., Bell N.C., Azoulay J.D., Bonizzoni M. (2019). A Sensor Array for the Discrimination of Polycyclic Aromatic Hydrocarbons Using Conjugated Polymers and the Inner Filter Effect. Chem. Sci..

[24] Yang Z., Fan L., Fan X., Hou M., Cao Z., Ding Y., Zhang W. (2020). Porphyrin-GO Nanocomposites Based NIR Fluorescent Sensor Array for Heparin Sensing and Quality Control. Anal. Chem..

[25] Ding Y., Wang J., Wang R., Xie Y. (2024). Development of Porphyrin-Based Fluorescent Sensors and Sensor Arrays for Saccharide Recognition. Chin. Chem. Lett..

[26] Yuan Y., Zhang L.L., Liu J.T., Zhang H.B., Xu J. (2021). Analysis and prediction of quality markers of *Lonicerae japonicae* Flos. China J. Chin. Mater. Medica.

[27] Valdes-García J., Zamora-Moreno J., Salomón-Flores M.K., Martínez-Otero D., Barroso-Flores J., Yatsimirsky A.K., Bazany-Rodríguez I.J., Dorazco-González A. (2023). Fluorescence Sensing of Monosaccharides by Bis-Boronic Acids Derived from Quinolinium Dicarboxamides: Structural and Spectroscopic Studies. J. Org. Chem..

[28] Basiruddin S., Swain S.K. (2016). Phenylboronic acid functionalized reduced graphene oxide based fluorescence nano sensor for glucose sensing. Mater. Sci. Eng. C.

[29] Cordes D.B., Gamsey S., Sharrett Z., Miller A., Thoniyot P., Wessling R.A., Singaram B. (2005). 18.The Interaction of Boronic Acid-Substituted Viologens with Pyranine: The Effects of Quencher Charge on Fluorescence Quenching and Glucose Response. Langmuir.

[30] Yu X., Fu L., Wang T., Liu Z., Niu N., Chen L. (2024). Multivariate Chemical Analysis: From Sensors to Sensor Arrays. Chin. Chem. Lett..

[31] Li X., Guo Z., Luo G., Miao P. (2022). Fluorescence DNA Switch for Highly Sensitive Detection of miRNA Amplified by Duplex-Specific Nuclease. Sensors.

[32] Yu Y., Ni W., Shi X., Bian Y., Li H., Liu M., Chen W., Zhang M., Jiang S., Cheng M. (2024). A Supramolecular Fluorescent Sensor Array Composed of Conjugated Fluorophores and Cucurbit[7]uril for Bacterial Recognition. Anal. Chem..

[33] Chen L., Tian X., Li Y., Yang C., Lu L., Zhou Z., Nie Y. (2019). An AIE Dye Based Smartphone and LDA Integrated Portable, Intelligent and Rapid Detection System as Trace Water Indicator and Cyanide Detector. Dye. Pigment..

[34] Liu Y., Minami T., Nishiyabu R., Wang Z., Anzenbacher P. (2013). Sensing of Carboxylate Drugs in Urine by a Supramolecular Sensor Array. J. Am. Chem. Soc..

[35] Li J., Qiao C., Liu H., Zhao D., Zhang J., Lu L., Huo D., Hou C. (2023). Fluorescence Nanoparticle Sensor Array Combined with Multidimensional Data Processing for the Determination of Small Organics and the Identification of *Baijiu*. Anal. Lett..

