# Supplementary material for: A Diboronic Acid-Based Fluorescent Sensor Array for Rapid Identification of Lonicerae Japonicae Flos and Lonicerae Flos
Source: Molecules. 2024 Sep 14;29(18):4374. doi: 10.3390/molecules29184374 (PMC11433768; doi:10.3390/molecules29184374)
Supplement: Supplementary file 1 [file molecules-29-04374-s001.zip › molecules-3164528-supplementary.pdf]

Supporting Information

A Diboronoic Acid-Based Fluorescent Sensor Array for Rapid Identification of Lonicerae Japonicae Flos and Lonicerae Flos

Ying Bian[1†], Chen-Qing Xing[1†], Yi Xu[2], Rong-Ping Zhu[2], Shuang-Lin Qin[3*], and Zhi-Jun Zhang[1*]

[1] School of Pharmacy, Hubei University of Science and Technology, Xianning 437100, China
[2] Xianning Public Inspection and Testing Center, Xianning, 437100 China
[3] Research Center for Precision Medication of Chinese Medicine, FuRong Laboratory, Hunan University of Chinese Medicine, Changsha 410000, China

Table of contents

1. The ^{1}H NMR spectrum of compound **Q1–Q3** ....Figure S1–S3

2. Statistical analysis.. Figure S4, Table S1–S9

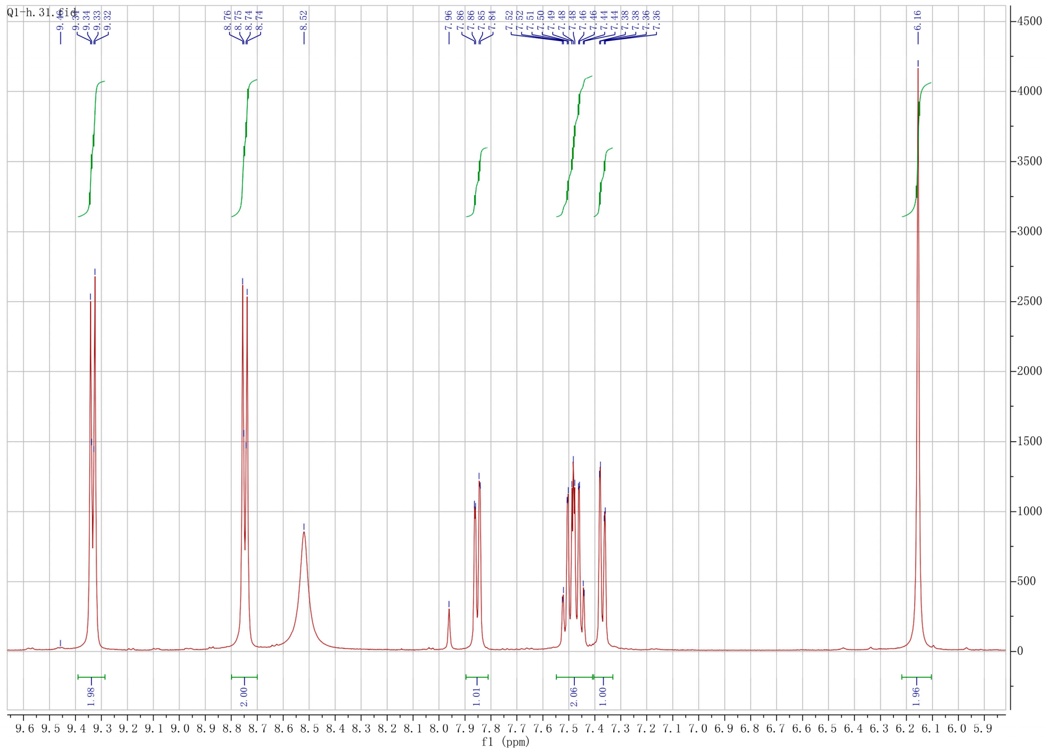

Figure S1. The ^{1}H NMR spectrum of compound **Q1** (400 MHz, DMSO-d_6)

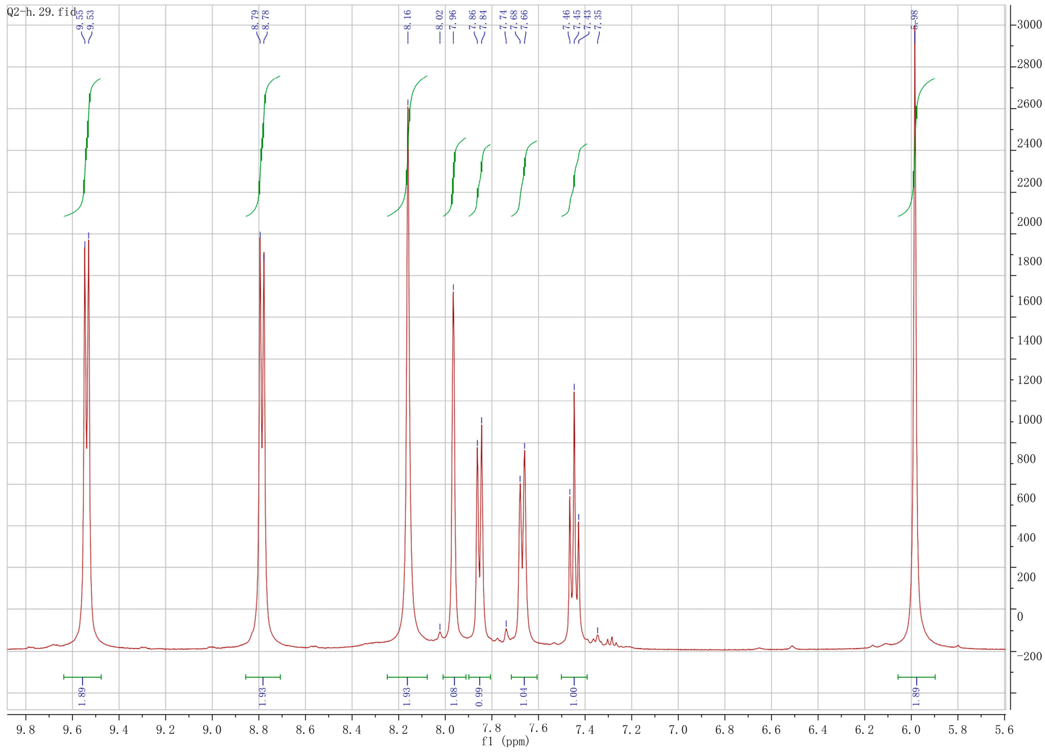

Figure S2. The ^{1}H NMR spectrum of compound **Q2** (400 MHz, DMSO-d_6)

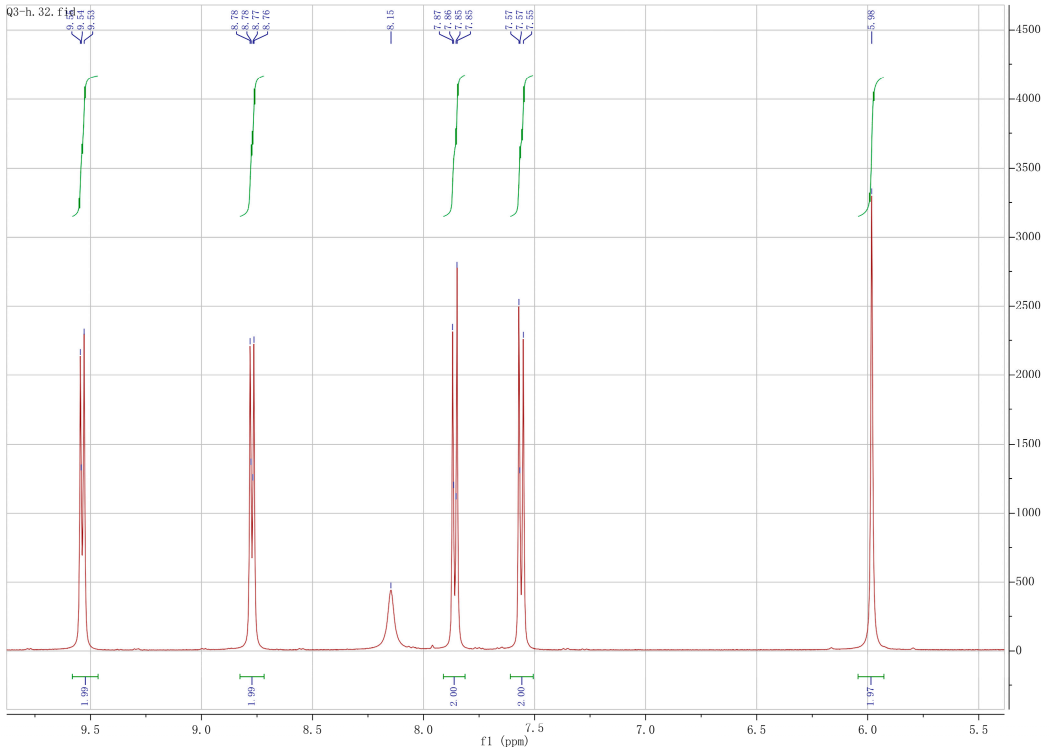

Figure S3. The ^{1}H NMR spectrum of compound **Q3** (400 MHz, DMSO-d_6)

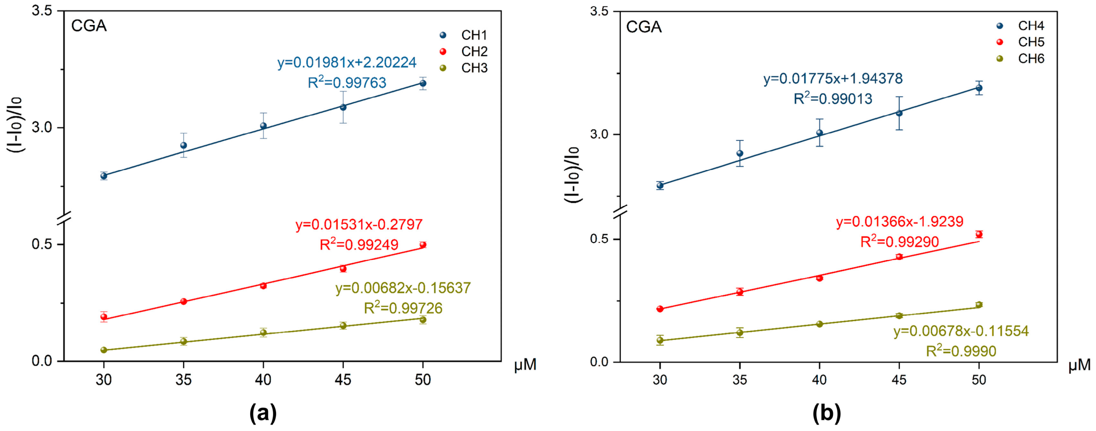

Figure S4. Calibration curves of the fluorescence responses to 5 different concentrations of CGA obtained from the sensor array in PBS (pH 7.2-7.4). (a) Fluorescence responses generated by channel 1 to channel 3 (CH1–CH3). (b) Fluorescence responses generated by channel 4 to channel 6 (CH4–CH6).

Table S1 Training matrix of fluorescence response pattern from an array of channel 1-channel 6 (CH 1-CH 6) against 4 polyphenols (50 μM) in PBS (pH 7.2-7.4). LDA was carried out and resulted in 3 factors of the canonical scores.

Analyte	Fluorescence response pattern						Result LDA			Group
polyphenols	CH1	CH2	CH3	CH4	CH5	CH6	F1	F2	F3	
CA	1.50	0.16	0.08	1.62	0.14	0.17	34.27	-28.51	0.25	1
CA	1.57	0.18	0.09	1.65	0.19	0.20	35.00	-25.32	0.19	1
CA	1.54	0.17	0.08	1.64	0.17	0.18	34.44	-26.85	-0.04	1
CA	1.49	0.16	0.08	1.59	0.12	0.12	33.56	-26.92	-1.30	1
CA	1.55	0.17	0.09	1.65	0.19	0.19	34.48	-26.50	-0.32	1
CA	1.46	0.16	0.07	1.57	0.08	0.11	33.38	-27.32	0.11	1
CGA	2.11	0.51	0.03	1.93	0.45	0.04	27.09	30.21	-0.48	2
CGA	2.04	0.51	0.03	1.88	0.34	-0.01	26.37	30.00	1.31	2
CGA	2.13	0.53	0.04	2.00	0.47	0.07	27.77	31.15	0.25	2
CGA	2.11	0.51	0.03	1.93	0.45	0.04	27.09	30.21	-0.48	2
CGA	2.12	0.52	0.04	1.93	0.46	0.06	26.64	30.51	0.28	2
CGA	2.09	0.51	0.03	1.91	0.43	0.03	26.48	30.19	-0.71	2
IAA	2.12	1.06	0.51	2.17	1.03	0.70	-30.96	-0.48	-1.94	3
IAA	2.25	1.11	0.59	2.27	1.13	0.80	-32.15	-1.36	-3.12	3
IAA	2.27	1.11	0.59	2.32	1.15	0.83	-31.17	-1.36	-2.53	3
IAA	2.21	1.08	0.54	2.18	1.09	0.75	-31.68	0.38	-3.22	3
IAA	2.27	1.16	0.59	2.33	1.22	0.85	-35.55	-0.41	-3.45	3
IAA	2.25	1.10	0.59	2.21	1.10	0.80	-32.91	-1.26	-2.12	3
IAC	2.18	1.05	0.54	2.19	0.99	0.75	-28.13	-1.29	0.82	4
IAC	2.29	1.10	0.58	2.23	1.08	0.90	-29.56	-1.57	4.24	4
IAC	2.26	1.06	0.57	2.21	1.08	0.88	-27.77	-3.49	3.17	4
IAC	2.20	1.05	0.55	2.20	1.06	0.82	-28.67	-3.67	0.91	4
IAC	2.23	1.06	0.56	2.20	1.07	0.88	-28.62	-4.89	3.57	4
IAC	2.28	1.09	0.57	2.21	1.08	0.89	-29.39	-1.43	4.60	4

Table S2 LDA jackknifed classification matrix table obtained from the array of channel 1-channel 6 against 4 polyphenols (50 μM) in PBS (pH 7.2-7.4). The jackknifed classification matrix with cross-validation reveals a 91.5% accuracy.

Analyte polyphenols	CA	CGA	IAA	IAC	%Correct
CA	6	0	0	0	100
CGA	0	6			100
IAA	0	0	5	1	83
IAC	0	0	1	5	83
Total	6	6	6	6	91.5

Table S3 Identification of unknown samples in PBS using LDA from the array of channel 1-channel 6. According to the verification, 15 among 16 unknown samples were correctly identified, representing an accuracy of 93.75%.

Analyte	Fluorescence response pattern						Result LDA			Gro	Identifi-	Verifica
Unknown	CH1	CH2	CH3	CH4	CH5	CH6	F1	F2	F3	up	cation	-tion
1	1.58	0.19	0.09	1.66	0.20	0.21	33.71	-24.66	0.50	1	CA	CA
2	1.58	0.18	0.09	1.66	0.20	0.20	34.73	-25.39	0.25	1	CA	CA
3	1.36	0.13	0.06	1.41	0.07	0.08	28.31	-32.93	-2.88	1	CA	CA
4	1.46	0.15	0.06	1.43	0.07	0.11	30.11	-27.61	-0.16	1	CA	CA
5	1.94	0.48	0.02	1.69	0.28	-0.02	22.01	24.06	0.94	2	CGA	CGA
6	2.04	0.49	0.03	1.86	0.29	-0.01	27.88	29.86	3.18	2	CGA	CGA
7	2.16	0.54	0.05	2.04	0.51	0.14	28.76	29.98	2.59	2	CGA	CGA
8	2.22	0.55	0.07	2.08	0.52	0.15	29.98	32.11	2.77	2	CGA	CGA
9	2.30	1.16	0.60	2.34	1.24	0.85	-36.23	0.54	-4.54	3	IAA	IAA
10	2.32	1.23	0.62	2.35	1.28	0.86	-41.30	3.76	-4.44	3	IAA	IAA
11	1.97	1.01	0.47	2.02	0.81	0.67	-29.57	-4.83	5.26	4	IAA	IAC
12	2.00	1.05	0.50	2.06	1.01	0.70	-35.72	-7.30	-2.84	3	IAA	IAA
13	2.31	1.11	0.60	2.25	1.09	0.92	-29.63	-1.71	4.75	4	IAC	IAC
14	1.93	0.93	0.49	1.94	0.90	0.72	-29.45	-17.24	-1.13	4	IAC	IAC
15	2.32	1.12	0.62	2.26	1.10	0.93	-31.10	-3.00	3.91	4	IAC	IAC
16	2.15	1.04	0.54	2.16	0.95	0.74	-27.84	-2.31	1.72	4	IAC	IAC

Table S4 Training matrix of fluorescence response pattern from an array of channel 1-channel 6 (CH 1-CH 6) against 14 baches of LJF and LF samples in PBS (pH 7.2-7.4). LDA was carried out and resulted in 3 factors of the canonical scores.

Analyte	Fluorescence response pattern						Results LDA			Group
LJF/LF	CH1	CH2	CH3	CH4	CH5	CH6	F1	F2	F3	
LJF-1	2.36	1.19	0.71	3.19	1.75	1.44	17.75	9.52	6.83	1
LJF-1	2.35	1.17	0.68	3.14	1.75	1.43	16.08	8.79	7.77	1
LJF-1	2.32	1.14	0.68	3.05	1.68	1.43	13.73	9.46	7.40	1
LJF-1	2.41	1.20	0.73	3.19	1.75	1.44	17.31	11.94	5.64	1
LJF-1	2.34	1.17	0.68	3.09	1.70	1.43	14.87	10.11	7.12	1
LJF-1	2.32	1.14	0.66	3.04	1.67	1.42	14.38	9.43	7.63	1
LJF-2	3.00	1.59	0.95	3.71	2.02	1.48	33.16	33.16	-3.61	2
LJF-2	3.06	1.60	0.97	3.72	2.02	1.49	33.86	36.11	-3.89	2
LJF-2	2.97	1.56	0.90	3.65	1.97	1.43	34.90	32.19	-3.47	2
LJF-2	2.98	1.59	0.94	3.70	1.99	1.48	33.43	32.75	-3.57	2
LJF-2	2.97	1.56	0.91	3.68	1.98	1.44	35.46	31.94	-3.25	2
LJF-2	2.98	1.58	0.94	3.70	1.98	1.48	33.61	32.58	-3.50	2
LJF-3	2.78	1.38	0.76	3.37	1.78	1.34	33.21	26.62	0.55	3
LJF-3	2.80	1.41	0.80	3.47	1.82	1.38	34.29	26.57	0.69	3
LJF-3	2.78	1.38	0.78	3.43	1.79	1.38	34.19	26.23	2.17	3
LJF-3	2.81	1.41	0.81	3.47	1.83	1.38	34.16	26.87	0.59	3

LJF-3	2.85	1.41	0.81	3.51	1.84	1.39	36.47	28.01	2.09	3
LJF-3	2.77	1.37	0.76	3.36	1.78	1.32	33.37	26.20	-0.18	3
LJF-4	2.23	1.15	0.76	3.03	1.67	1.41	8.22	9.49	-0.66	4
LJF-4	2.23	1.11	0.75	3.01	1.65	1.41	8.76	9.67	0.88	4
LJF-4	2.23	1.11	0.74	3.01	1.57	1.41	12.02	10.33	2.02	4
LJF-4	2.28	1.16	0.76	3.06	1.69	1.42	9.77	10.82	0.58	4
LJF-4	2.30	1.18	0.76	3.06	1.71	1.44	8.44	11.23	0.95	4
LJF-4	2.38	1.19	0.76	3.06	1.71	1.44	9.99	14.71	1.70	4
LJF-5	2.11	1.14	0.73	1.93	1.76	1.57	-63.25	18.51	-10.73	5
LJF-5	2.12	1.17	0.76	2.02	1.78	1.57	-60.29	18.05	-11.74	5
LJF-5	2.11	1.12	0.72	1.93	1.76	1.57	-62.03	17.84	-9.07	5
LJF-5	2.11	1.11	0.71	1.92	1.76	1.56	-62.39	17.82	-9.28	5
LJF-5	2.13	1.21	0.77	2.04	1.78	1.60	-61.20	18.60	-12.37	5
LJF-5	2.12	1.14	0.75	1.94	1.77	1.57	-63.63	18.89	-11.49	5
LJF-6	2.00	1.05	0.65	1.95	1.72	1.86	-66.48	9.78	12.34	6
LJF-6	1.95	1.00	0.64	1.90	1.67	1.80	-65.53	9.22	10.69	6
LJF-6	1.96	1.00	0.64	1.91	1.69	1.81	-65.84	9.25	11.21	6
LJF-6	2.09	1.09	0.66	1.96	1.73	1.86	-65.45	13.79	12.26	6
LJF-6	1.99	1.05	0.65	1.93	1.71	1.85	-67.65	9.93	11.80	6
LJF-6	1.99	1.02	0.65	1.92	1.69	1.81	-65.24	10.14	10.83	6
LF-1	1.83	0.85	0.51	2.69	1.49	1.22	8.06	-8.14	5.01	7
LF-1	1.87	0.87	0.52	2.76	1.52	1.29	9.00	-7.84	8.66	7
LF-1	1.84	0.86	0.51	2.71	1.49	1.23	8.47	-8.33	5.70	7
LF-1	1.88	0.88	0.54	2.78	1.53	1.33	7.41	-7.16	10.00	7
LF-1	1.86	0.86	0.52	2.72	1.52	1.25	7.61	-7.48	6.43	7
LF-1	1.87	0.87	0.53	2.77	1.53	1.32	7.80	-7.77	9.94	7
LF-2	1.61	0.77	0.43	2.47	1.38	1.10	2.50	-14.93	-0.32	8
LF-2	1.63	0.79	0.44	2.49	1.40	1.12	1.55	-14.44	-0.05	8
LF-2	1.62	0.77	0.44	2.49	1.39	1.12	2.42	-14.81	0.59	8
LF-2	1.61	0.77	0.42	2.45	1.36	1.08	2.55	-14.63	-1.07	8
LF-2	1.64	0.79	0.45	2.52	1.42	1.15	2.50	-14.53	1.86	8
LF-2	1.64	0.81	0.46	2.53	1.43	1.16	1.07	-14.32	1.09	8
LF-3	1.52	0.74	0.42	2.49	1.33	1.12	3.87	-18.54	1.72	9
LF-3	1.54	0.76	0.42	2.50	1.34	1.13	3.19	-18.06	1.35	9
LF-3	1.51	0.73	0.42	2.46	1.31	1.10	3.14	-18.22	0.35	9
LF-3	1.51	0.73	0.42	2.46	1.30	1.08	4.25	-18.28	-0.50	9
LF-3	1.56	0.77	0.43	2.50	1.34	1.13	3.17	-17.18	1.34	9
LF-3	1.50	0.73	0.39	2.45	1.29	1.07	5.09	-18.82	0.23	9
LF-4	1.60	0.76	0.39	2.40	1.25	0.99	7.83	-13.44	-3.72	10
LF-4	1.61	0.76	0.41	2.41	1.27	1.00	6.87	-12.50	-4.28	10
LF-4	1.63	0.79	0.43	2.46	1.30	1.05	6.06	-12.44	-3.48	10
LF-4	1.61	0.76	0.41	2.41	1.27	1.00	6.87	-12.50	-4.28	10
LF-4	1.62	0.76	0.42	2.43	1.27	1.04	6.54	-12.35	-2.47	10

LF-4	1.62	0.78	0.43	2.43	1.30	1.04	5.01	-12.36	-3.53	10
LF-5	1.61	0.81	0.45	2.45	1.37	1.05	1.59	-13.76	-5.74	11
LF-5	1.62	0.82	0.45	2.48	1.40	1.06	1.93	-14.16	-5.64	11
LF-5	1.58	0.81	0.42	2.45	1.35	1.03	2.65	-15.09	-5.92	11
LF-5	1.57	0.80	0.39	2.39	1.32	1.01	2.44	-15.35	-5.55	11
LF-5	1.65	0.83	0.46	2.50	1.40	1.06	3.09	-12.76	-5.68	11
LF-5	1.57	0.80	0.39	2.39	1.32	1.01	2.44	-15.35	-5.55	11
LF-6	1.55	0.78	0.40	2.48	1.34	1.07	4.46	-17.79	-1.09	12
LF-6	1.59	0.81	0.43	2.60	1.45	1.12	5.24	-18.97	0.73	12
LF-6	1.60	0.81	0.44	2.60	1.45	1.14	4.65	-18.10	0.93	12
LF-6	1.58	0.80	0.43	2.60	1.44	1.09	6.62	-19.00	-0.47	12
LF-6	1.58	0.79	0.41	2.52	1.37	1.07	5.63	-17.29	-1.10	12
LF-6	1.58	0.79	0.41	2.52	1.37	1.07	5.67	-17.61	-1.09	12
LF-7	1.70	0.88	0.44	2.63	1.42	1.14	8.07	-13.91	0.99	13
LF-7	1.64	0.78	0.41	2.53	1.36	1.09	7.77	-14.87	1.97	13
LF-7	1.67	0.83	0.42	2.61	1.41	1.11	9.58	-15.42	2.06	13
LF-7	1.64	0.78	0.41	2.53	1.36	1.09	7.77	-14.87	1.97	13
LF-7	1.64	0.78	0.39	2.51	1.35	1.07	8.33	-14.87	1.22	13
LF-7	1.65	0.81	0.41	2.61	1.40	1.11	10.31	-16.10	3.14	13
LF-8	1.66	0.88	0.50	2.44	1.34	1.13	-2.62	-9.06	-7.38	14
LF-8	1.68	0.90	0.51	2.44	1.34	1.13	-2.83	-8.23	-7.96	14
LF-8	1.65	0.88	0.49	2.41	1.31	1.12	-3.05	-8.81	-7.40	14
LF-8	1.63	0.84	0.47	2.37	1.27	1.11	-2.26	-9.08	-6.20	14
LF-8	1.64	0.85	0.48	2.41	1.30	1.11	-1.51	-9.28	-6.00	14
LF-8	1.70	0.91	0.52	2.48	1.35	1.13	-1.26	-7.75	-7.72	14

Table S5 Identification of unknown LJF and LF samples in PBS using LDA from the array of channel3 1-channel 6. According to the verification, 55 among 56 unknown samples were correctly identified, representing an accuracy of 98.21%.

Analyte	Fluorescence response patter						Results LDA			Gro	Identifi-	Verifica-
Unknown	CH1	CH2	CH3	CH4	CH5	CH6	F1	F2	F3	up	cation	tion
1	2.29	1.11	0.63	2.96	1.63	1.34	17.75	9.52	6.83	1	LJF-1	LJF-1
2	2.29	1.11	0.64	3.01	1.66	1.41	16.08	8.79	7.77	1	LJF-1	LJF-1
3	2.43	1.26	0.74	3.22	1.77	1.47	13.73	9.46	7.40	1	LJF-1	LJF-1
4	2.41	1.22	0.74	3.20	1.76	1.44	17.31	11.94	5.64	1	LJF-1	LJF-1
5	3.07	1.67	0.99	3.79	2.04	1.50	14.87	10.11	7.12	2	LJF-2	LJF-2
6	2.94	1.50	0.88	3.63	1.96	1.41	14.38	9.43	7.63	2	LJF-2	LJF-2
7	3.06	1.66	0.97	3.72	2.04	1.49	33.16	33.16	-3.61	2	LJF-2	LJF-2
8	2.97	1.56	0.89	3.64	1.97	1.43	33.86	36.11	-3.89	2	LJF-2	LJF-2
9	2.74	1.35	0.75	3.34	1.77	1.30	34.90	32.19	-3.47	3	LJF-3	LJF-3
10	2.89	1.45	0.81	3.53	1.89	1.41	33.43	32.75	-3.57	3	LJF-3	LJF-3
11	2.49	1.35	0.74	3.31	1.77	1.29	35.46	31.94	-3.25	3	LJF-3	LJF-3
12	2.85	1.44	0.81	3.51	1.85	1.39	33.61	32.58	-3.50	3	LJF-3	LJF-3
13	2.41	1.23	0.83	3.12	1.77	1.46	33.21	26.62	0.55	4	LJF-4	LJF-4

14	2.38	1.22	0.77	3.07	1.72	1.45	34.29	26.57	0.69	4	LJF-4	LJF-4
15	2.21	1.08	0.74	2.97	1.57	1.32	34.19	26.23	2.17	4	LJF-4	LJF-4
16	2.17	0.79	0.68	2.79	1.55	1.32	34.16	26.87	0.59	4	LJF-4	LJF-4
17	2.15	1.22	0.81	2.05	1.81	1.62	36.47	28.01	2.09	5	LJF-5	LJF-5
18	2.04	1.07	0.71	1.92	1.74	1.56	33.37	26.20	-0.18	5	LJF-5	LJF-5
19	2.23	1.23	0.84	2.06	1.82	1.64	8.22	9.49	-0.66	5	LJF-5	LJF-5
20	2.02	1.04	0.69	1.91	1.73	1.55	8.76	9.67	0.88	5	LJF-5	LJF-5
21	1.93	0.94	0.61	1.84	1.66	1.74	12.02	10.33	2.02	6	LJF-6	LJF-6
22	1.94	0.99	0.62	1.88	1.66	1.76	9.77	10.82	0.58	6	LJF-6	LJF-6
23	2.12	1.10	0.66	1.97	1.74	1.87	8.44	11.23	0.95	6	LJF-6	LJF-6
24	2.13	1.10	0.69	1.99	1.75	1.89	9.99	14.71	1.70	6	LJF-6	LJF-6
25	1.80	0.84	0.49	2.66	1.46	1.20	-63.25	18.51	-10.73	7	LF-1	LF-1
26	1.75	0.84	0.47	2.63	1.43	1.19	-60.29	18.05	-11.74	7	LF-1	LF-1
27	1.93	0.90	0.57	2.80	1.54	1.34	-62.03	17.84	-9.07	7	LF-1	LF-1
28	1.92	0.88	0.54	2.79	1.53	1.34	-62.39	17.82	-9.28	7	LF-1	LF-1
29	1.59	0.74	0.39	2.42	1.31	1.05	-61.20	18.60	-12.37	8	LF-2	LF-2
30	1.70	0.85	0.49	2.60	1.44	1.18	-63.63	18.89	-11.49	8	LF-2	LF-2
31	1.68	0.81	0.49	2.55	1.44	1.18	-66.48	9.78	12.34	8	LF-2	LF-2
32	1.60	0.76	0.41	2.43	1.36	1.08	-65.53	9.22	10.69	8	LF-2	LF-2
33	1.56	0.78	0.43	2.53	1.35	1.14	-65.84	9.25	11.21	9	LF-3	LF-3
34	1.48	0.70	0.36	2.37	1.23	1.02	-65.45	13.79	12.26	9	LF-3	LF-3
35	1.56	0.80	0.44	2.58	1.36	1.14	-67.65	9.93	11.80	9	LF-3	LF-3
36	1.49	0.71	0.36	2.41	1.24	1.06	-65.24	10.14	10.83	9	LF-3	LF-3
37	1.59	0.75	0.39	2.38	1.24	0.98	8.06	-8.14	5.01	10	LF-4	LF-4
38	1.65	0.84	0.45	2.51	1.35	1.09	9.00	-7.84	8.66	10	LF-4	LF-4
39	1.65	0.81	0.44	2.50	1.34	1.08	8.47	-8.33	5.70	10	LF-4	LF-4
40	1.56	0.75	0.37	2.32	1.17	0.97	7.41	-7.16	10.00	10	LF-4	LF-4
41	1.55	0.79	0.38	2.39	1.32	0.98	7.61	-7.48	6.43	11	LF-5	LF-5
42	1.67	0.89	0.46	2.51	1.41	1.07	7.80	-7.77	9.94	11	LF-5	LF-5
43	1.65	0.86	0.46	2.51	1.41	1.06	2.50	-14.93	-0.32	11	LF-5	LF-5
44	1.54	0.79	0.36	2.38	1.31	0.98	1.55	-14.44	-0.05	11	LF-5	LF-5
45	1.61	0.83	0.46	2.62	1.47	1.15	2.42	-14.81	0.59	12	LF-6	LF-6
46	1.55	0.78	0.38	2.46	1.34	1.07	2.55	-14.63	-1.07	12	LF-6	LF-6
47	1.53	0.74	0.34	2.45	1.32	1.06	2.50	-14.53	1.86	12	LF-6	LF-6
48	1.60	0.83	0.45	2.60	1.46	1.15	1.07	-14.32	1.09	12	LF-6	LF-6
49	1.70	0.88	0.47	2.64	1.42	1.17	3.87	-18.54	1.72	13	LF-7	LF-7
50	1.63	0.78	0.39	2.49	1.34	1.06	3.19	-18.06	1.35	13	LF-7	LF-7
51	1.62	0.77	0.32	2.48	1.34	1.05	3.14	-18.22	0.35	13	LF-7	LF-7
52	1.70	0.92	0.47	2.68	1.43	1.17	4.25	-18.28	-0.50	13	LF-7	LF-7
53	1.63	0.84	0.46	2.32	1.26	1.10	3.17	-17.18	1.34	14	LF-8	LF-8
54	1.70	0.91	0.53	2.48	1.36	1.14	5.09	-18.82	0.23	14	LF-8	LF-8
55	1.71	0.92	0.54	2.49	1.37	1.15	7.83	-13.44	-3.72	14	LF-8	LF-8
56	1.59	0.81	0.34	2.27	1.25	1.08	6.87	-12.50	-4.28	11	LF-8	LF-4

Table S6 LDA jackknifed classification matrix table obtained from the array of channel 1-channel 6 against 14 baches of LJF and LF samples. The jackknifed classification matrix with cross-validation reveals a 100% accuracy.

Analyte	LJF-1-	LJF-2	LJF-3	LJF-4	LJF-5	LJF-6	LF-1	LF-2	LF-3	LF-4	LF-5	LF-6	LF-7	LF-8	%Correct
LJF-1	6	0	0	0	0	0	0	0	0	0	0	0	0	0	0
LJF-2	0	6	0	0	0	0	0	0	0	0	0	0	0	0	100
LJF-3	0	0	6	0	0	0	0	0	0	0	0	0	0	0	100
LJF-4	0	0	0	6	0	0	0	0	0	0	0	0	0	0	100
LJF-5	0	0	0	0	6	0	0	0	0	0	0	0	0	0	100
LJF-6	0	0	0	0	0	6	0	0	0	0	0	0	0	0	100
LF-1	0	0	0	0	0	0	6	0	0	0	0	0	0	0	100
LF-2	0	0	0	0	0	0	0	6	0	0	0	0	0	0	100
LF-3	0	0	0	0	0	0	0	0	6	0	0	0	0	0	100
LF-4	0	0	0	0	0	0	0	0	0	6	0	0	0	0	100
LF-5	0	0	0	0	0	0	0	0	0	0	6	0	0	0	100
LF-6	0	0	0	0	0	0	0	0	0	0		6	0	0	100
LF-7	0	0	0	0	0	0	0	0	0	0	0	0	6	0	100
LF-8	0	0	0	0	0	0	0	0	0	0	0	0	0	6	100
Total	6	6	6	6	6	6	6	6	6	6	6	6	6	6	100

Table S7 Training matrix of fluorescence response pattern from an array of channel 1-channel 6 (CH 1-CH 6) against Jinyinhua Mixtures from 3 different manufacturers in PBS (pH 7.2-7.4). LDA was carried out and resulted in 2 factors of the canonical scores.

Analyte	Fluorescence response pattern						Results LDA		Group
Mixtures	CH1	CH2	CH3	CH4	CH5	CH1	F1	F2	
M1	1.56	0.71	0.18	1.94	0.83	0.45	1.72	5.40	1
M1	1.57	0.71	0.19	1.94	0.84	0.45	1.57	5.33	1
M1	1.55	0.68	0.17	1.92	0.81	0.44	2.12	6.02	1
M1	1.55	0.68	0.18	1.92	0.83	0.44	1.44	5.85	1
M1	1.53	0.64	0.16	1.90	0.81	0.44	1.61	6.17	1
M1	1.53	0.65	0.16	1.91	0.81	0.44	1.85	5.64	1
M2	1.29	0.57	0.07	1.66	0.73	0.34	12.79	-2.77	2
M2	1.29	0.58	0.07	1.67	0.74	0.35	9.73	-3.89	2
M2	1.28	0.57	0.06	1.65	0.73	0.34	12.52	-3.10	2
M2	1.25	0.56	0.04	1.64	0.72	0.33	12.04	-4.40	2
M2	1.27	0.56	0.05	1.64	0.72	0.34	12.27	-2.69	2
M2	1.25	0.56	0.04	1.62	0.69	0.33	12.56	-3.81	2
M3	1.58	0.73	0.18	2.05	0.91	0.53	-14.26	-0.99	3
M3	1.54	0.71	0.16	1.98	0.89	0.51	-12.93	-0.82	3
M3	1.55	0.72	0.17	2.03	0.90	0.52	-11.99	-2.24	3
M3	1.49	0.68	0.13	1.96	0.89	0.51	-14.05	-3.97	3
M3	1.59	0.74	0.18	2.06	0.92	0.54	-15.74	-1.32	3
M3	1.50	0.70	0.14	1.97	0.89	0.51	-13.28	-4.40	3

Table S8 Identification of unknown Jinyinhua Mixtures samples in PBS using LDA from the array of channel 1-channel 6. According to the verification, 12 among 12 unknown samples were correctly identified, representing an accuracy of 100%.

Analyte	Fluorescence response patter						Results LDA		Group	Identifi-cation	Verifica-tion
Unknown	CH1	CH2	CH3	CH4	CH5	CH6	F1	F2			
1	1.59	0.72	0.19	1.98	0.84	0.45	1.41	5.89	1	M1	M1
2	1.61	0.74	0.21	2.00	0.85	0.46	1.99	6.94	1	M1	M1
3	1.50	0.63	0.15	1.89	0.78	0.42	3.64	4.18	1	M1	M1
4	1.50	0.63	0.15	1.90	0.80	0.43	3.07	3.86	1	M1	M1
5	1.30	0.58	0.07	1.69	0.74	0.36	9.34	-3.36	2	M2	M2
6	1.31	0.59	0.09	1.70	0.75	0.37	8.08	-4.16	2	M2	M2
7	1.24	0.50	0.04	1.61	0.69	0.30	16.91	-1.39	2	M2	M2
8	1.25	0.53	0.04	1.61	0.69	0.32	13.87	-1.98	2	M2	M2
9	1.47	0.68	0.06	1.96	0.88	0.51	-15.66	-4.02	3	M3	M3
10	1.61	0.76	0.18	2.07	0.93	0.55	-16.39	0.14	3	M3	M3
11	1.65	0.76	0.20	2.11	0.93	0.55	-15.54	1.89	3	M3	M3
12	1.44	0.67	0.05	1.96	0.85	0.51	-15.39	-6.80	3	M3	M3

Table S9 LDA jackknifed classification matrix table obtained from the array of channel 1-channel 6 against Jinyinhua Mixtures from 3 different manufacturers in PBS (pH 7.2-7.4). The jackknifed classification matrix with cross-validation reveals a 100% accuracy.

Analyte Mixtures	M1	M2	M3	%Correct
M1	6	0	0	100
M2	0	6	0	100
M3	0	0	6	100
Total	6	6	6	100
